# Work–Life Balance: The True Failure Is in Not Trying

**DOI:** 10.3389/fped.2016.00037

**Published:** 2016-04-28

**Authors:** Denise Rita Purdie

**Affiliations:** ^1^Pediatric Critical Care, University of California Los Angeles, Los Angeles, CA, USA

**Keywords:** work–life balance, well-being, medical education, mentoring, residency training

*Defeat is not the worst of failures*.

*Not to have tried is the true failure*.

George Edward Woodberry

Last year, I was one of two chief residents at a mid-sized Pediatric residency program. We both started out the academic year excited for the opportunity to bridge the gap between the residents and the faculty/administration. For our roles as educators and administrators – those responsibilities detailed in the “job description” – we felt well prepared. However, a surprising proportion of our time was spent trying to protect the well-being of our residents. It was through that effort that I was introduced to the concept of work–life balance.

Work–life balance is a relatively new concept in the field of medicine and is considered by many to be a generational desire. Younger physicians are often cited as demanding that their professional lives make a little room for their personal lives. The concepts of flexible schedules, part-time hours, and job sharing are the fruits of their labor. Well-being and mindfulness are the new maxims in medical schools and training programs. This sea change in the culture of medicine, however, has not been universally applauded nor accepted by those we consider our mentors.

A regular New York Times Op Ed contributor, the anesthesiologist Karen S. Sibert, MD, argued that, “Medicine shouldn’t be a part-time interest to be set aside if it becomes inconvenient; it deserves to be a life’s work (1).” This attitude pervades academic medicine (2). Indeed, this has been the culture of medicine for hundreds of years. Physicians were always available. Residents and faculty alike would spend days on end in the hospital. They were ready and willing to miss soccer games and school plays, anniversaries, and holidays to be of service to their patients.

However, this no longer appears to be tenable for many physicians whether at the trainee or the faculty level. The first few weeks of my year as chief resident were punctuated by the suicides of two interns training at programs in New York. The number of trainees at my own program who were deeply affected by those deaths surprised me. They were saddened by – but unfortunately understood very well – the struggles endured by those two interns at the beginning of their residency. They empathized with those trainees and with those that remained.

An insightful Op Ed written by an Internal Medicine resident at Yale entitled, “Why Do Doctors Commit Suicide,” appeared in the New York Times in response to those tragedies. In the article, he reminds the reader of the enthusiasm with which we begin our residencies, and the “fatigue, emotional exhaustion, and crippling self-doubt” that unfortunately follow for many trainees (3). An exponential increase in the workload from medical school into residency contributes to that transformation. Suddenly, the intern is expected to care for twice to three times as many patients, remember their patients’ complex medical histories, and understand the evidence upon which their patients’ care is based.

This is certainly true at my former program – a well-respected, high-volume children’s hospital. The interns spend the majority of their morning pre-rounding, rounding, calling consults, and putting in orders. The remainder of the day is relegated to finishing their progress notes, answering an inexplicable number of pages, and putting in more orders. The cumbersome nature of notes, multiplicity of information sources, and lack of clarity writing orders drags the days’ tasks well into the evening. They are expected to (and definitely would like to) return to their homes and dedicate the remainder of their waking hours to reading about their patients’ conditions. Instead, they prep their notes for the following day and update discharge summaries until they finally end up in bed with 4–5 h of sleep ahead.

During these task-driven days, unfortunately, there are fewer opportunities for formal education. Morning report has been whittled down to a mere 30 min in the era of duty hour restrictions. Rounds, given the great number of patients and a relatively truncated day, are necessarily succinct. Afternoon teaching sessions with the fellows or attendings are unattended because of patient responsibilities. A caesura in the day’s madness for self-directed learning – reading a textbook, reviewing the literature – seldom occurs.

Despite these barriers, my former program produces well-trained physicians. The benefits of experiential learning in such a high-volume environment, in the presence of excellent physicians, cannot be denied. However, the tragedy of dwindling time for formal education is obvious. The years of experience that these physicians have accumulated could provide invaluable lessons for the residents. Understaffed academic hospitals with overworked clinician-educators are the locales in which this demanding workday milieu exists to the detriment of education. I have observed a similar situation at my current program. I have heard the same stories from friends training around the country.

As a former chief resident, the toll this takes on the mental and physical well-being of trainees is unacceptable. Residents feel guilty because they cannot possibly spend any more time reading. They feel guilty because they do not spend enough time with their patients. They feel guilty when their exhausted attendings ask them to see “just one more patient” when their team is capped. They feel guilty because they do not see their friends, they do not talk to their families, and they were not able to travel home for the holidays. They have not bought groceries, done the laundry, or walked their dogs for weeks.

Poor nutrition, dehydration, and minimal exercise are the hallmarks of many physicians’ lives (4, 5). According to one study, only 38% of resident physicians have a personal family physician. More alarming is that 25% of those with chronic illnesses and 40% who use prescription medications regularly do not have a physician (6). My co-chief and I often had to force febrile, ill-appearing residents to make doctors’ appointments despite the clear effect their illness was having on their ability to safely provide patient care. Physicians, especially trainees, lead lives marked by imbalance despite that maxim to “heal thyself.”

This imbalance has considerable long-term consequences. Work–life imbalance has been associated with decreased job satisfaction, productivity, and eventual burnout. As Shanafelt et al. summarized in their national survey evaluating burnout among physicians, “burnout may erode professionalism, influence quality of care, increase the risk for medical errors, and promote early retirement (7).” And the incidence of physician burnout is steadily increasing. According to another survey, from 2013 to 2015, physician burnout increased from 39.8 to 46% (8). Studies have shown that physician rates of suicidal ideation and suicide may be higher than in the general population (9–12).

These are depressing trends within a field defined by altruism. My colleagues often tell me that they would not choose medicine again if given the opportunity. They do not recommend the life of a physician to younger relatives and family friends. I witnessed more than a few incredible clinician-educators battle with unappreciated long hours in the hospital. One-by-one they retired from the field of medicine – they became stay-at-home parents, pharmaceutical consultants, and research scientists. We are losing/we will lose the smartest and the brightest if we are unwilling to evolve in this profession. Those who are willing to sacrifice their personal life in favor of work are commendable, but this should no longer be the expectation.

Many in my generation would not be willing to “suck it up” the way that older physicians were willing to. Rather than fighting that reality, we should accept this new culture of medicine. True, our incomes may suffer in the era of part-time hours and job sharing. We may have to rely on advanced practice providers such as physician assistants and nurse practitioners to cover the expanding “ranks of insured patients (1).” I recognize that I am in the infancy of my career – with my limited experience I cannot possibly imagine the myriad ramifications of this shift in medicine. I understand that many are concerned that these changes may negatively affect patient care. However, patient care has already been shown to suffer when work–life balance does not exist.

There are physicians that have achieved what some have deemed the “ephemeral.” For those of my generation whose well-being requires some “balance” (because there are plenty that do not), these individuals might serve as mentors. Fledgling mentoring programs have been shown to improve aspects of “job-related well-being, self-esteem, and self-efficacy” in early academics in some institutions (13). This is not surprising given that medical education’s foundation has traditionally been that of apprenticeship. Mentoring and role modeling are not exceptional ideas in our profession – the future might just require some adjustment in focus to maintain happy and healthy physicians.

I would like to end by thanking my former residency program for realizing the magnitude of this issue. The associate program director and program director supported the residents and chief residents in developing a physician well-being/work–life balance curriculum. Program funds have been allocated to a number of activities, including dedicated monthly noon conferences, resident-developed evening sessions, and a quarterly book club at an attendings’ home. Resident daytime and nighttime patient caps were decreased to protect patient safety and resident education. While these endeavors are in their infancy, they are a step in the right direction.

Residency is an ideal time for future physicians to start thinking about their need for work–life balance. It would be wonderful if academic institutions supported mentoring programs and well-being curricula to help their trainees (and physicians) in this regard. Our current and future trainees deserve at least the opportunity to maintain balance in their lives.

## Author Contributions

The author confirms being the sole contributor of this work and approved it for publication.

## Conflict of Interest Statement

The author declares that the research was conducted in the absence of any commercial or financial relationships that could be construed as a potential conflict of interest.
